# Overexpression of the *tcp* Gene Cluster Using the T7 RNA Polymerase/Promoter System and Natural Transformation-Mediated Genetic Engineering of *Vibrio cholerae*

**DOI:** 10.1371/journal.pone.0053952

**Published:** 2013-01-07

**Authors:** Sandrine Borgeaud, Melanie Blokesch

**Affiliations:** Global Health Institute, School of Life Sciences, Ecole Polytechnique Fédérale de Lausanne, Lausanne, Switzerland; University of Osnabrueck, Germany

## Abstract

The human pathogen and aquatic bacterium *Vibrio cholerae* belongs to the group of naturally competent bacteria. This developmental program allows the bacterium to take up free DNA from its surrounding followed by a homologous recombination event, which allows integration of the transforming DNA into the chromosome. Taking advantage of this phenomenon we genetically engineered *V. cholerae* using natural transformation and FLP recombination. More precisely, we adapted the T7 RNA polymerase/promoter system in this organism allowing expression of genes in a T7 RNA polymerase-dependent manner. We naturally transformed *V. cholerae* by adding a T7-specific promoter sequence upstream the toxin-coregulated pilus (*tcp*) gene cluster. In a *V. cholerae* strain, which concomitantly produced the T7 RNA polymerase, this genetic manipulation resulted in the overexpression of downstream genes. The phenotypes of the strain were also in line with the successful production of TCP pili. This provides a proof-of-principle that the T7 RNA polymerase/promoter system is functional in *V. cholerae* and that genetic engineering of this organism by natural transformation is a straightforward and efficient approach.

## Introduction

A plethora of methods exist today to allow genetic engineering of bacteria. Many of those methods have been used for the last 30 years or even longer and straightforward protocols are available today to master those techniques. One prominent example is the seminal book *Molecular Cloning: A Laboratory Manual* published initially in 1982 by Tom Maniatis, Edward Fritsch, and Joseph Sambrook [1] (the currently available 4^th^ edition is authored by Michael R. Green and Joseph Sambrook; http://www.molecularcloning.com). But even though many techniques are available and work well, reducing the time needed for cloning is often desired. Thus, we developed a fast protocol [2]–[4] to genetically modify our favorite organism, the human pathogen and aquatic bacterium *Vibrio cholerae*, using chitin-induced natural transformation [5], [6].

**Figure 1:**
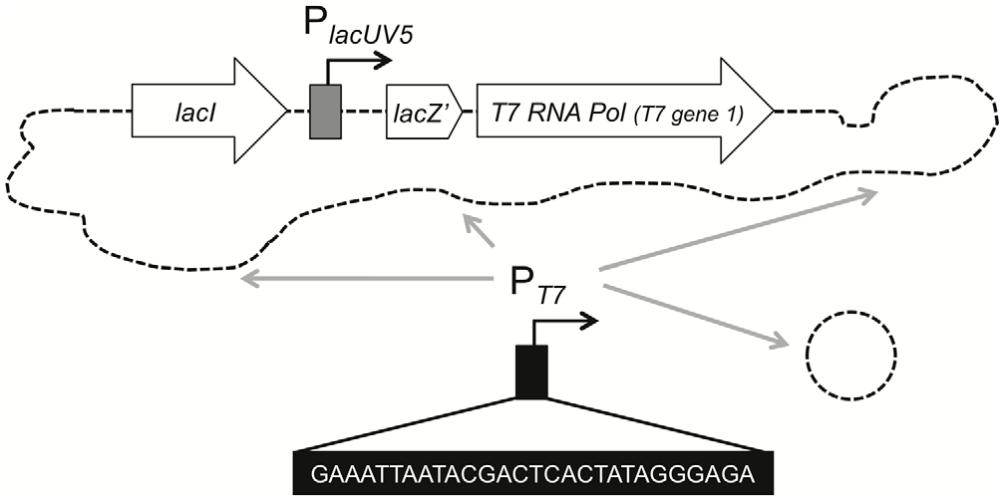
Schematic representation of the experimental setup. The rational behind this study was to engineer *V. cholerae* strains so that large gene clusters can be artificially expressed independent of growth condition restraints. The idea was to integrate the lacUV5-promoter controlled T7 RNA polymerase-encoded gene together with its repressor gene (*lacI*; both derived from *E. coli* BL21(DE3) [12]) into the *V. cholerae* chromosome. Using this strain, specific genes could be put under control of the T7 RNA polymerase solely by integrating the T7 RNA polymerase-dependent promoter sequence (indicated in black box; P_T7_) at the respective locus on the chromosome using natural transformation. Alternatively, the P_T7_ sequence was integrated on a plasmid as is commonly done in *E. coli* overexpression systems.

The regulatory network of chitin-induced natural transformation of *V. cholerae* is extremely complicated and brings together the pathways of chitin sensing and degradation, quorum sensing, and carbon catabolite repression (summarized in [7], [8] and recently reviewed in [9]). However, the advantage of the DNA uptake process of *V. cholerae* over some other naturally competent Gram-negative bacteria such as *Haemophilus influenzae* and *Neisseria gonorrhoeae* is that *V. cholerae* is not fastidious about the kind of DNA it takes up while it is competent [10]. This makes natural competence and transformation a perfect system for fast and efficient delivery of DNA to the cells. In this study we applied our previously published protocol describing the combination of natural transformation and FLP recombination in *V. cholerae* (TransFLP, [3], [4]) in order to integrate DNA sequences in a site-specific manner onto the chromosome. The rational behind this study was to create *V. cholerae* strains in which we were able to artificially express large gene clusters. We reasoned that it would be difficult or even impossible to clone such gene clusters onto plasmids/cosmids thereby expressing the genes *in trans*. Furthermore, as many of the common plasmids seem to be quickly lost from *V. cholerae* without constant selection pressure plasmid-encoded expression was excluded from this study. Consequently, we decided to express our gene cluster of interest by adding a strong and specific promoter *in cis* of the indigenous genes on the chromosome using our TransFLP method (Fig. 1).

To our knowledge the strongest and most exclusive expression system described so far in bacteria is based on the T7 RNA polymerase/promoter combination. Several studies in the 1980's have led to the establishment of this very successful system [11], [12], which is still used our days (mostly with variations from the original protocols). The gene encoding the T7 DNA-directed RNA polymerase (EC 2.7.7.6) (from here on referred to as T7 RNA polymerase), phage T7 *gene 1*, was first cloned by Davanloo *et al*. in 1984 [13]. This opened up new possibilities as the T7 RNA polymerase “has a stringent specificity for its own promoters and will selectively transcribe DNA that has been linked to such a promoter” [13]. Such specificity was considered useful for directing the expression of only a subset of genes within a cell. The T7 RNA polymerase-dependent promoter is a “highly conserved sequence of 23 continuous base pairs including the start site for the RNA” [13]. Few if any of these conserved sequences are found in bacterial hosts causing this stringent specificity of the enzyme. The 22–23 base pairs consensus sequence of the T7 RNA polymerase promoter has been previously studied [14]–[16]. However, Ikeda *et al*. identified the essential bases of this specific promoter sequence by screening a randomly mutagenized library of T7 promoter sequences and correlating the *in vivo* activity to the sequences [17]. In our study the T7 Φ10 promoter was used, which encompasses 28 bp: GAAATTAATACGACTCACTATAGGGAGA [17], [18]. We choose this strong expression system to have the best possible outcome as our study is aimed at proof of principle. Below we explain the cloning strategy and the experimental execution in detail. Furthermore, we provide evidence that the target gene cluster was indeed highly expressed in the genetically engineered strain, which resulted in the desired phenotypes. Obstacles and future needs for improvement are also discussed.

The target of our first trial was the toxin-coregulated pilus (*tcp*) [19] gene cluster of *V. cholerae*. The *tcp* gene cluster is located on the *Vibrio* pathogenicity island 1 (VPI-1) [20] previously termed TCP-ACF element [21]. The reason for this choice was that this cluster has been extensively studied *in vivo* and also under virulence inducing conditions *in vitro* (e.g. through the use of AKI growth conditions [22], [23]). The TCP pilus is essential for intestinal colonization as it allows the establishment of microcolonies in the host [19], which was shown both in humans and in the infant mouse animal model of cholera [24]–[27]. The bundle-forming TCP pilus is a member of Type IVb pili, which “are found almost exclusively on enteric bacteria” [28]. The TCP pilus (classical and El Tor type) has been characterized based on mutant analysis, electron microscopy (EM; such as TEM, SEM, STEM, and three-dimensional high-resolution field emission scanning electron microscopy, FESEM), crystallization of the major pilin TcpA, and CryoEM reconstruction of full length TCP [28]–[32]. Based on *in vivo* and *in vitro* data it was concluded that a major function of this pilus is to mediate interactions between bacteria through microcolony formation that is required for intestinal colonization [30]. Indeed, Kirn *et al*. provided evidence that autoagglutination correlates well with intestinal colonization in the infant mouse model [30]. Thus, we hypothesized that induced expression of the *tcp* cluster would provide us with phenotypes such as *in vitro* autoagglutination/microcolony formation due to TCP production. It should be noted that artifical induction of the *tcp* cluster was already performed earlier by expressing *toxT*, which encodes the major transcriptional activator of virulence genes in *V. cholerae* [33], from an inducible promoter [29]. This method led to the production of the major subunit of TCP, TcpA, both in the classical and El Tor biotypes of *V. cholerae*. Furthermore, TCP fibers of *V. cholerae* O1 biotype El Tor could be visualized by EM upon *toxT* overexpression [29]. However, whereas autoagglutination of the classical biotype strain was readily observable under such artificial induction conditions, autoagglutination of a *V. cholerae* O1 El Tor strain (C6706) did not occur [31]. The author of this study speculated that the reduced level of TcpA protein and TCP fibers (∼50%) for the O1 El Tor biotype compared to the classical strain O395 could partially explain this lack of autoagglutination [31]. To circumvent this issue, we aimed at high-level expression of the whole *tcp* cluster by directly inducing this cluster using the T7 RNA polymerase dependent system described above. The promoter sequence was thereby introduced upstream of the *tcpA* gene in a site-directed manner using the natural transformation-based TransFlp method as a tool [3], [4]. Proof-of-principle was provided by successful TCP-mediated agglutination, microcolony formation, and the visualization of TCP fibers using scanning electron microscopy (SEM).

## Materials and Methods

### Bacterial strains and plasmids

*V. cholerae* strains and plasmids used in this study are indicated in Table 1. *E. coli* strains DH5α [34] and BL21(DE3) [12] were used as hosts for cloning purposes and to test T7 RNA polymerase-dependent expression constructs. Bacterial mating between *V. cholerae* and *E. coli* was done using *E. coli* strain S17-1λpir [35] as the donor strain.

**Table 1:** Bacterial strains and plasmids.

Strains or plasmids	Genotype*	References
***V. cholerae*** ** strains**		
A1552	Wild type, O1 El Tor Inaba, Rif^R^	[42]
A1552-GFP	A1552 with mTn7-gfp, Rif^R^, Gm^R^	[36]
AT7RNAP	A1552 containing mTn7-T7RNAP, Rif^R^ Gm^R^	this study
AΔctxAB	A1552ΔctxAB::FRT, Rif^R^	this study
AΔctxAB-T7RNAP	A1552ΔctxAB::FRT containing mTn7-T7RNAP, Rif^R^ Gm^R^	this study
AΔctxAB-[P_T7_]-tcp	A1552ΔctxAB::FRT, [P_T7_]-tcp::FRT, Rif^R^	this study
AΔctxAB-[P_T7_]-tcp-T7RNAP	A1552ΔctxAB::FRT, [P_T7_]-tcp::FRT, containing mTn7-T7RNAP, Rif^R^ Gm^R^	this study
**Plasmids**		
pBR-Tet_MCSI	pBR322 derivative deleted for Tet promoter and part of *tet* ^R^ gene; Amp^R^	[7]
pBR-[P_T7_]-GFP	*gfp* gene preceded by T7 RNA polymerase-dependent promoter sequence; Amp^R^	this study
pUX-BF13	oriR6K, helper plasmid with Tn7 transposition function; Amp^R^	[37]
pGP704::Tn7	pGP704 with mini-Tn7	[41]
pGP704-mTn7-T7RNAP	pGP704 with mini-Tn7 carrying *lacI* and P*_lacUV5_*-driven T7 DNA-directed RNA polymerase gene; Amp^R^	this study

* VC numbers according to [47].

### Media and growth conditions

Overnight cultures of *V. cholerae* and *E. coli* strains were grown in LB medium with shaking at 30°C. Bacterial strains used for gene expression profiling, phenotypic characterization and SEM were also cultured in LB either in the absence or in the presence of 1 mM IPTG. Ampicillin was added for plasmid maintenance at a concentration of 100 μg/ml whenever required. Fifty μg/ml gentamicin was used to select mTn7-T7RNAP-containing *V. cholerae* cells after bacterial mating with *E. coli* cells. *E. coli* cells were counter-selected by using *Vibrio* selective medium (TCBS; Fluka).

### Construction of mini-Tn7 transposon carrying the gene encoding for T7 RNA polymerase

Plasmid pGP704-mTn7-T7RNAP was generated by ligating the *Sma*I-digested vector pGP704::Tn7 (Table 1) with the *lacI*-P_lacUV_-T7RNAP cluster-containing and *Sca*I-digested PCR fragment. PCR was performed using primer pair LacI-before and T7 RNA pol after ( Table S1) and genomic DNA of *E. coli* strain BL21(DE3) as template [12]. The construct was verified by sequencing.

### Construction of T7 RNA polymerase-dependent transcriptional reporter plasmid

Plasmid pBR-[P_T7_]-GFP was constructed as follows. The *gfp* coding region (including the Shine-Dalgarno sequence) was PCR-amplified using oligonucleotides P[T7]-GFP-up-P and P[T7]-GFP-down-*Bam*HI (Table S1) and genomic DNA of strain A1552-GFP [36] as template. The upstream primer had a 5′ phosphorylated and non-priming overhang containing the T7 RNA polymerase dependent promoter sequence (as indicated in Fig. 1). The fragment was digested with *Bam*HI and cloned into *Eco*RV/*Bam*HI double-digested vector pBR-Tet_MCSI [7]. Correct ligation was validated by colony PCR and sequencing.

### Construction of *Vibrio cholerae* strains

*V. cholerae* strains carrying the gene encoding T7 RNA polymerase on the chromosome were created by triparental mating between the respective *V. cholerae* strain (Table 1), *E. coli* strain S17λpir/pUX-BF13 [37], and *E. coli* strain S17λpir/pGP704-mTn7-T7RNAP. The latter suicide plasmid consists of vector pGP704 as backbone and the mini-Tn7 transposon [38] containing the gene cluster *lacI*-P_lacUV_-T7RNAP as cargo. Mini-Tn7 transposons (mTn7) insert site-specifically and at a neutral site in Gram-negative bacteria including *V. cholerae* [38], [39].

The T7 RNA polymerase-dependent promoter sequence was site-specifically integrated into the chromosome using natural transformation and FLP recombination (TransFLP method; [3], [4]). Oligonucleotides for the design of the transforming PCR fragment are indicated in Table S1. The *ctx* operon was likewise deleted (using PCR fragment ΔctxAB-FRT-Kan-FRT; primer indicated in Table S1).

### Quantitative reverse transcription PCR (qRT-PCR)

*V. cholerae* strains were grown for several hours in LB containing 1mM IPTG until they reached an optical density at 600 nm of ∼2. IPTG-induced strain AΔctxAB-[P_T7_]-tcp-T7RNAP was harvested at the same time though the OD_600_ values varied due to extensive clumping of the cells (autoagglutination as described in the result section). Harvesting of cells, RNA preparation, reverse transcription and qPCR were performed as previously described [7]. Expression levels were normalized to the housekeeping gene *gyrA*. Primers used for qRT-PCR are listed in Table S1.

### Epifluorescence microscopy

Visualization of bacterial cells at the microscopic level was done using a Zeiss Axio Imager M2 epifluorescence microscope. Image acquisition was performed using the Zeiss AxioVision software steering a high-resolution camera (AxioCam MRm). Images were rotated, cropped and magnified using Zeiss AxioVision and ImageJ.

### Scanning electron microscopy (SEM)

Bacterial cells were grown as described. At the time of harvest, cells were spotted onto a silicon wafer. Attachment was allowed to occur for several minutes before the cells were fixed for 1 hour with 1.25% glutaraldehyde and 1% tannic acid in 0.1 M phosphate buffer, pH 7.4. Cells were washed in phosphate buffered saline and then further fixed for 30 minutes in 1.0% osmium tetroxide in the same buffer. The bacteria, attached to the wafers, were dehydrated in a graded alcohol series and then dried by passing through the supercritical point of carbon dioxide (Leica Microsystems CPD300). The samples were coated with a 2 nm layer of osmium using an osmium plasma coater (Filgen OPC60). Images of the bacteria were taken with a field emission scanning electron microscope (Merlin, Zeiss NTS) using an acceleration voltage of 2 kV and the in-lens secondary electron detector.

## Results and Discussion

The aim of this study was to genetically engineer *V. cholerae* strains so that large gene clusters could be artificially expressed *in vitro*. Consequently, the experimental design was as follows (Fig. 1): First, we integrated the T7 RNA polymerase encoding gene (*T7 gene 1*) preceded by the lacUV5 promoter and the Lac repressor gene (*lacI*) into the *V. cholerae* chromosome. The T7 specific promoter sequence was delivered on a plasmid (control) as well as upstream the *tcp* operon. The latter was accomplished using chitin-induced natural transformation followed by FLP recombination. Expression of *tcp* and expected phenotypes were confirmed.

### Integration of T7 RNA polymerase encoding gene into the *V. cholerae* chromosome

As the T7 RNA polymerase has a very stringent specificity towards the T7-specific promoter sequences [40] we decided to integrate the encoding gene (T7 *gene 1*; [13]) into the *V. cholerae* genome (Fig. 1). To do so we amplified the T7 *gene 1* preceded by the lacUV5 promoter and the lac repressor gene *lacI* from the λ(DE3) prophage contained in the lysogenic *E. coli* strain BL21(DE3) [12] using PCR and primers LacI-before and T7 RNA pol after (Table S1). After restriction enzyme digestion (see Material and Methods) the PCR fragment was ligated into the likewise digested miniTn7 transposon [37] harbored on plasmid pGP704::Tn7 [41]. After triparental mating between the *E. coli* strains S17-1λpir/pGP704-mTn7-T7RNAP (carrying the construct-containing transposon), S17-1λpir/pUX-BF13 (providing the Tn7 transposition *in trans* [37]), and *V. cholerae* wild type strain A1552 [42] the resulting *V. cholerae* strain AT7RNAP was selected on gentamicin-containing TCBS agar plates. Single colonies were isolated, grown on LB agar plates, and verified by colony PCR for the correct and site-directed integration of the transposon (data not shown).

### Functionality of T7 RNA polymerase in *V. cholerae*

**Figure 2:**
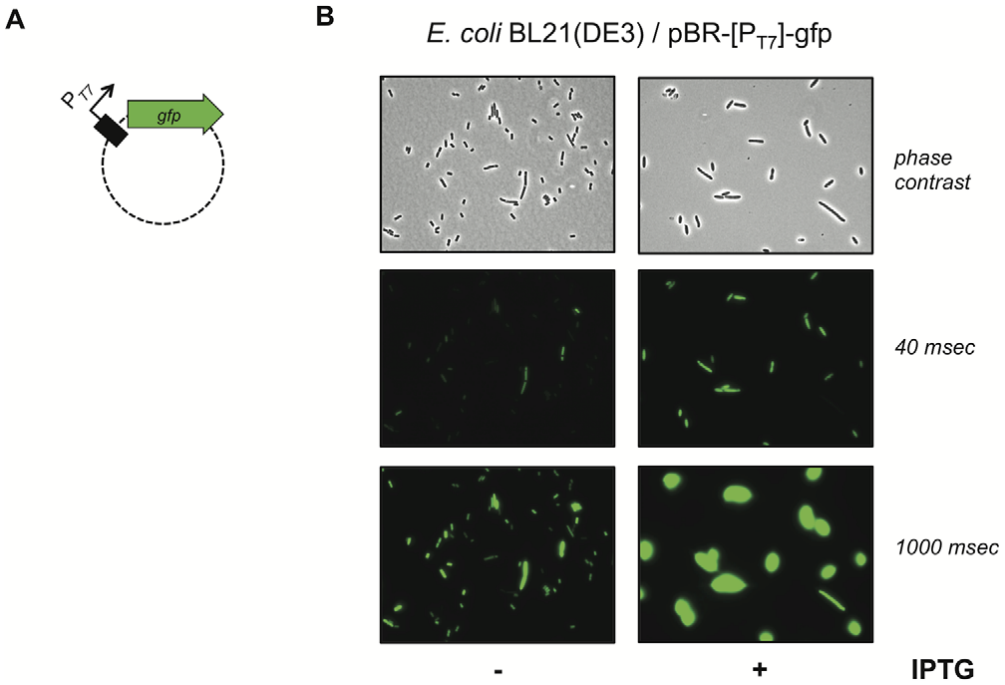
Functionality of T7 RNA polymerase dependent reporter plasmid. Plasmid pBR-[P_T7_]-GFP (panel A) was transferred into chemically competent *E. coli* BL21(DE3) cells. Transformed bacteria were grown in the absence or presence of 1 mM IPTG as indicated and tested for GFP expression using epifluorescence microscopy (panel B). Panel B upper row: phase contrast images showing all cells; middle row: green fluorescence channel with short exposure time (40 msec); lower row: green fluorescence channel with longer exposure time (1000 msec).

In order to test whether the T7 RNA polymerase gene was functional in *V. cholerae* a transcriptional reporter gene was cloned onto a plasmid (Table 1). To accomplish this, the green florescent protein-encoding gene (*gfp*) was PCR-amplified from *V. cholerae* strain A1552-GFP [36]. Concomitantly with this amplification step we added the T7 promoter sequence “GAAATTAATACGACTCACTATAGGGAGA” (Fig. 1) upstream the *gfp* coding region by incorporating this sequence as 5′-overhang into the forward primer (Table S1). The respective P_T7_-*gfp* fragment was ligated in the promoterless plasmid pBR-Tet_MCSI [7] yielding plasmid pBR-[P_T7_]-gfp (Table 1). The functionality of this plasmid was first tested in the well-characterized *E. coli* strain BL21(DE3) [12](Fig. 2) as this strain has been extensively used over the years for T7 RNA polymerase dependent high-level expression of proteins. The respective *E. coli* transformant was grown in rich medium either in the absence or presence of the inducer IPTG and T7 RNA polymerase-dependent expression was visualized using epifluorescence microscopy (Fig. 2). Though low-level expression of *gfp* was also observed under uninduced growth conditions (as discussed below), the expression was significantly enhanced upon provision of the inducer. Thus, the functionality and usefulness of the reporter plasmid was established.

**Figure 3:**
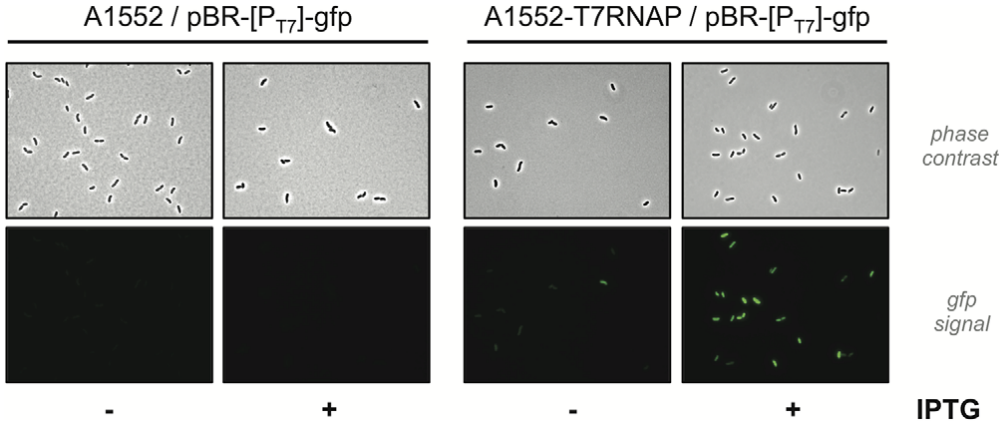
Testing for T7 RNA polymerase-dependent expression of *gfp* reporter construct in *V. cholerae*. Plasmid pBR-[P_T7_]-GFP was transferred into *V. cholerae* strain A1552 (WT; on the left) or its T7 RNA polymerase derivative AT7RNAP (on the right). Plasmid-containing bacteria were grown in rich medium either in the absence (−) or in the presence (+) of the inducer IPTG. Expression of *gfp* driven by the T7 RNA polymerase-dependent promoter was visualized by epifluorescence microscopy (green channel; lower row; same exposure time was applied to all samples). The corresponding phase contrast images are shown above.

Next, the reporter plasmid was transferred in two different *V. cholerae* strains: the wild type strain A1552 and the newly constructed strains AT7RNAP (T7RNAP^+^) (Fig. 3). Similar to the experiment described above for *E. coli*, we grew both transformants in rich medium without or with IPTG as the inducer of T7 RNA polymerase production. Whereas no green fluorescent signal was detectable under both conditions in the *V. cholerae* strain lacking T7 RNA polymerase (Fig. 3, left), *gfp* expression was unambiguously detectable in strain AT7RNAP and significantly enhanced in the presence of the inducer IPTG (Fig. 3, right). We conclude that the T7 RNA polymerase was functional in *V. cholerae* and that T7 RNA polymerase specific promoter sequences were not unspecifically transcribed (or at least not at high levels) in *V. cholerae* strains lacking T7 RNA polymerase. Thus, the system seemed feasible for induction of large gene clusters in a T7 RNA polymerase-dependent manner.

### Integration of the T7 RNA polymerase specific promoter sequence upstream *tcpA* using the TransFLP method

**Figure 4:**
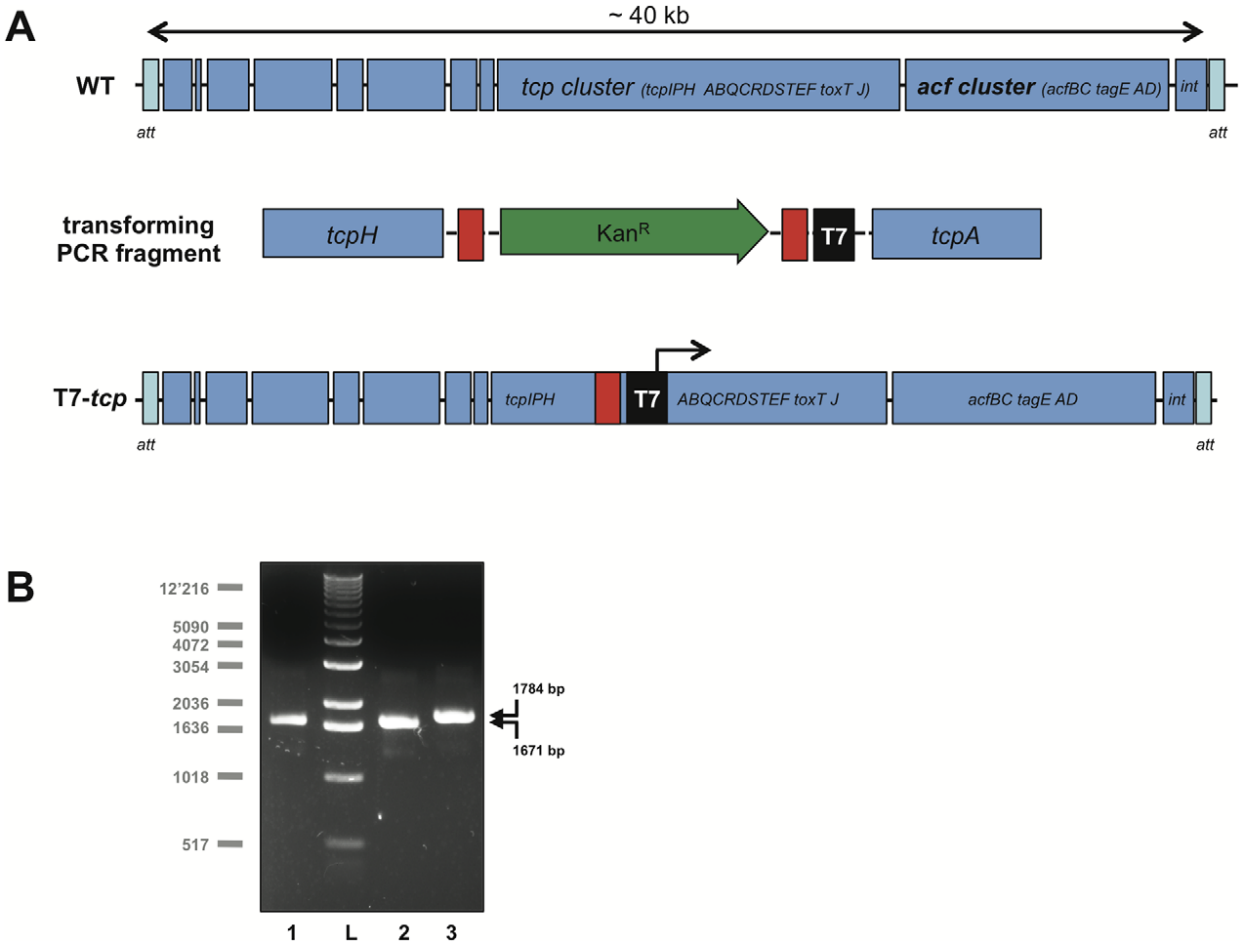
Insertion of the T7 RNA polymerase-dependent promoter sequence by TransFLP. A: Schematic representation depicting the strategy to integrate the T7 RNA polymerase-dependent promoter sequence into the *V. cholerae* chromosome. Upper row: the *Vibrio* pathogenicity island (VPI-1 or *tcp* island) is indicated for the WT strain of *V. cholerae* (freely adapted from [46] and based on [47]; not to scale). Middle row: the transforming PCR-derived DNA fragment included parts of the genes *tcpH* and *tcpA* as flanking regions (in blue) to allow homologous recombination with the chromosome. In addition the PCR fragment carried the FRT-site (red rectangles) flanked kanamycin resistant cassette (*aph*; green arrow) and the T7 RNA polymerase-dependent promoter sequence (black box; according to Fig. 1). Lower row: the structure of the VPI-1 island after natural transformation and FLP recombination of the WT strain using the PCR fragment indicated in the middle row as transforming DNA material. **B: PCR-based verification of site-directed insertion.** The T7 RNA polymerase-dependent promoter sequence was inserted into the *V. cholerae* genome by chitin-induced natural transformation followed by FLP-mediated excision of the antibiotics resistance cassette (TransFLP; [3], [4]). The correctness of the resulting strain was tested by PCR using primer pair T7tcp-chk-up & T7tcp-chk-down and genomic DNA as template. The expected fragment sizes for the wild type (A1552; lane 1) and the *ctx* minus parental strain (AΔctxAB-T7RNAP; lane 2) (both 1′671 bp in length) as well as for the newly created T7 RNA polymerase-dependent promoter-containing strain AΔctxAB-[PT7]-tcp-T7RNAP (lane 3; 1′784 bp) are indicated by arrows. L, 1 kb ladder (Invitrogen; sizes indicated on the left).

As described above and as a proof-of-principle we were interested in artificially expressing the *tcp* cluster *in vitro* under non-virulence inducing conditions. As it has previously been shown that induced expression of *toxT* led to enhanced TcpA and cholera toxin (CT) production [29] and as *toxT* is embedded within the *tcp* cluster (Fig. 4) we decided to first attenuate the newly constructed *V. cholerae* strain (AT7RNAP) by deleting the *ctxAB* operon using chitin-induced TransFLP (data not shown) [2]–[4]. The resulting strain was named AΔctxAB-T7RNAP (Table 1). Next, to create an artificial induction system for the *tcp* gene cluster we needed to integrate the T7 RNA polymerase specific promoter sequence (Fig. 1) upstream the first gene whose gene product is directly involved in the toxin-coregulated pilus biosynthesis, namely *tcpA* (Fig. 4A). We did so by creating a PCR fragment consisting of the following features (Fig. 4A, middle row): 1) an FRT-site flanked antibiotic resistance cassette (here *aph* adding kanamycin resistance to the cells); 2) flanking regions at both ends spanning *tcpH* and *tcpA* to allow double-homologous recombination; 3) the specific T7 RNA polymerase promoter sequence (black box stating T7 in Fig. 4A). This PCR fragment was obtained in two rounds of PCR as described earlier [3] and used as transforming DNA in a chitin-induced transformation assay [2]. The acceptor strain in this assay was AΔctxAB-T7RNAP (T7RNAP^+^). Transformants were selected on kanamycin-containing agar plates followed by the excision of the Kan^R^ cassette using FLP recombination as previously described [3]. The resulting *V. cholerae* strain, AΔctxAB-[P_T7_]-tcp-T7RNAP, was verified for the integration of the T7 RNA polymerase specific promoter sequence by PCR using purified genomic DNA as template (Fig. 4B).

### T7 RNA polymerase-dependent expression of *tcp* genes under non-virulence inducing conditions

**Figure 5:**
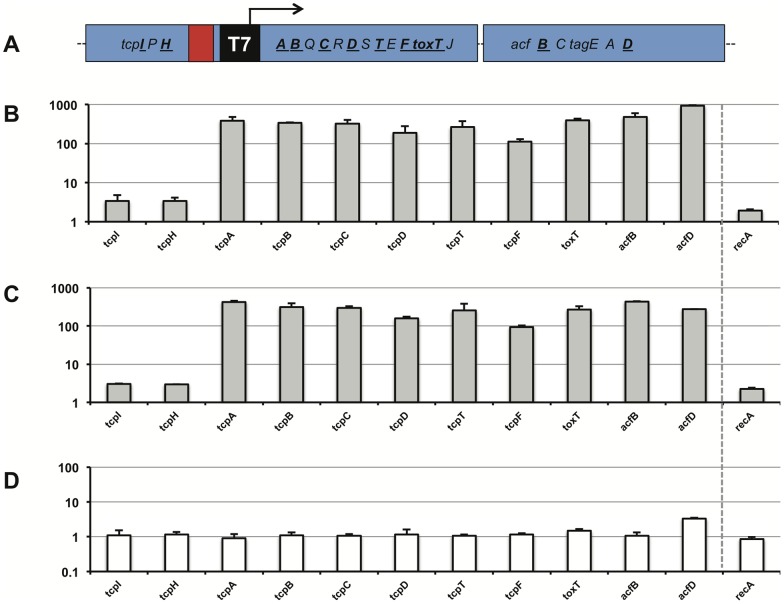
Expression of VPI-1 genes in a T7 RNA polymerase-dependent manner. Expression of genes upstream and downstream the T7 RNA polymerase dependent promoter sequence was tested using qRT-PCR. **A: Schematic representation of the genomic region of interest in the final strain AΔctxAB-[P_T7_]-tcp-T7RNAP.** Genes whose expression was tested by qRT-PCR in panels B–C are indicated in bold and underlined. The P_T7_ promoter is indicated as a black rectangle with a black arrow on top. **B–D: Comparison of gene expression between the engineered** ***V. cholerae*** **strain and its parental strains.** Gene expression was measured in the engineered *V. cholerae* strain containing both the T7 RNA polymerase gene and the P_T7_ promoter sequence and compared to its two parental strains each containing only one of both elements. Bacteria were grown in rich medium in the presence of 1 mM IPTG. Genes indicated on the X-axis were tested for their expression level using qRT-PCR (normalized to the housekeeping gene *gyrA*). The values shown on the Y-axis depict the relative expression difference between strains AΔctxAB-[P_T7_]-tcp-T7RNAP ([P_T7_]^+^, T7RNAP^+^) and AΔctxAB-[P_T7_]-tcp ([P_T7_]^+^, T7RNAP^−^)(panel B), between strains AΔctxAB-[P_T7_]-tcp-T7RNAP ([P_T7_]^+^, T7RNAP^+^) and AΔctxAB-T7RNAP ([P_T7_]^−^, T7RNAP^+^)(panel C), and between the two control strains AΔctxAB-T7RNAP ([P_T7_]^−^, T7RNAP^+^) and AΔctxAB-[P_T7_]-tcp ([P_T7_]^+^, T7RNAP^−^) (panel C), respectively. Relative expression levels of *recA* are shown for comparison reason. Average of two independent biological replicates.

The newly created strain AΔctxAB-[PT7]-tcp-T7RNAP ([P_T7_]^+^, T7RNAP^+^) as well as the parental strains lacking either the T7 *gene 1* (AΔctxAB-[P_T7_]-tcp) or lacking the T7 specific promoter sequence (AΔctxAB-T7RNAP) were grown under non-virulence inducing conditions. After harvesting the cells, RNA was extracted and qRT-PCR was performed (Fig. 5). The expression of diverse VPI-1 genes located either upstream (*tcpI* and *tcpH*) of the T7 specific promoter sequence or downstream of the latter (*tcpA,C,C,D,T,F, toxT, acfB,D*; Fig. 5) was determined. As depicted in Fig. 5 all genes located downstream of P_T7_ were at least 100-fold induced compared to either parental strain (Fig. 5B and C). In contrast the expression of genes located upstream of P_T7_ were similar to the expression measured for the parental strains and in the range of the expression that was also observed for a housekeeping gene (*recA*). Importantly, neither the T7 RNA polymerase nor the P_T7_ promoter alone was sufficient to induce *tcp* expression (Fig. 5D) in contrast to the strain containing both elements (Fig. 5B, C). We conclude that the T7 RNA polymerase/promoter system is able to drive *tcp* expression in *V. cholerae*.

### Phenotypes associated with T7 RNA polymerase-mediated *tcp* expression in *V. cholerae*

**Figure 6:**
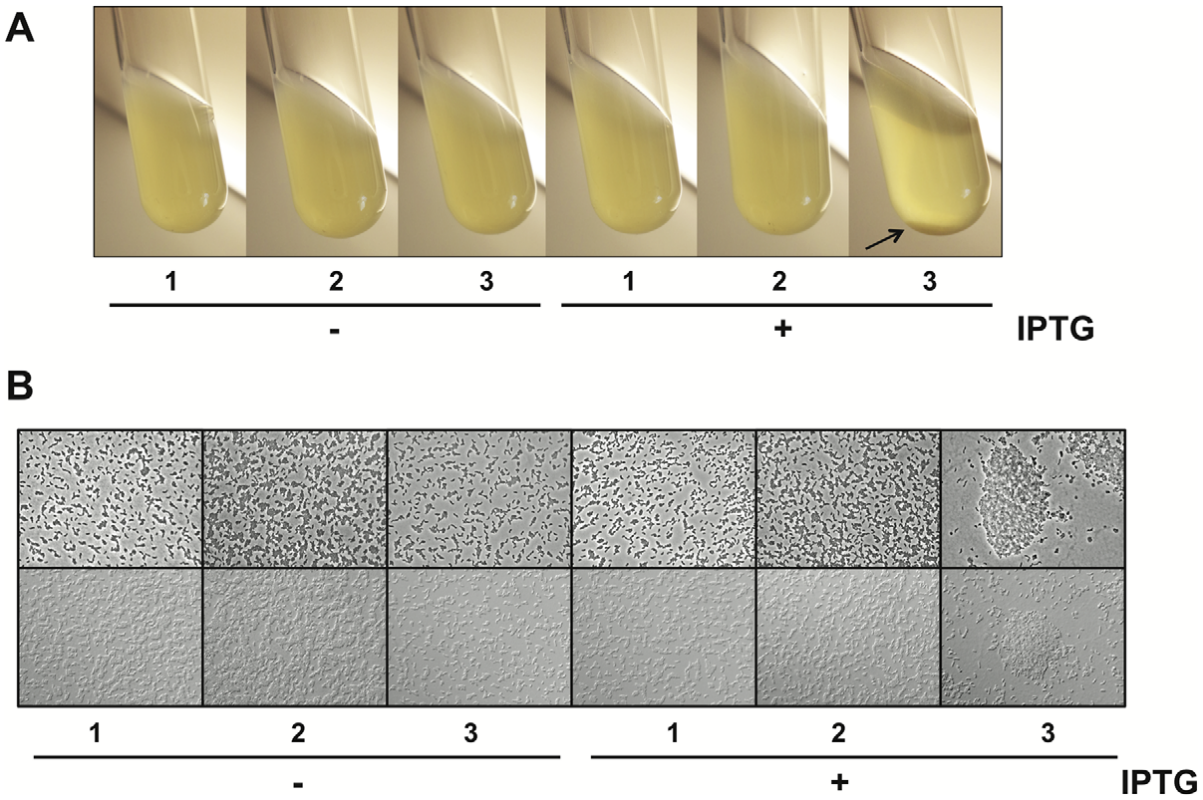
Phenotypes of T7 RNA polymerase-mediated expression of the *tcp* cluster. The *V. cholerae* strains AΔctxAB-[P_T7_]-tcp (lane 1), AΔctxAB-T7RNAP (lane 2), and AΔctxAB-[P_T7_]-tcp-T7RNAP (lane 3) were grown under shaking conditions in LB medium without or with supplementation of 1 mM IPTG. **A: Macroscopic observation of an autoagglutination phenotype.** Bacterial cultures were allowed to settle before pictures were taken. Agglutinated bacteria are indicated by a black arrow in the rightmost image. **B: Microscopic observation of a microcolony formation phenotype.** Microcolony formation of the cells was visualized using light microscope. Pictures were taken using phase contrast (upper panel) or DIC (lower panel).

Taylor *et al*. provided evidence that *in vitro* induction of TCP (under virulence-inducing conditions of *V. cholerae* biotype classical) resulted in autoagglutination of the bacteria [19]. Such autoagglutination led to the formation of macroscopic bacterial clumps and “large aggregates that collect rapidly at the bottom of the culture tube” [30]. Closer inspection of such clumps by SEM suggested that they are reminiscent of microcolonies formed upon intestinal colonization [30]. Furthermore, the authors of this study concluded that “the ability of TCP mutants to participate in microcolonies correlates with the ability of those strains to colonize the infant mouse” [30]. Consequently, we checked for autoagglutination of *V. cholerae* as an *in vitro* phenotype after P_T7_-driven expression of the *tcp* cluster (Fig. 6A). Indeed, autoagglutination was confirmed and fully dependent on the presence of the T7 RNA polymerase and the T7 RNA polymerase specific promoter sequence (Fig. 6A). In contrast, the parental strains harboring only one of those elements did not autoagglutinate. Furthermore, no cellular clumps were visible in the absence of autoinducer (-IPTG) even though we provided evidence that low expression also occured under those conditions (Fig. 3). This is consistent with the insufficient expression of *tcp* upon artificial *toxT* expression as discussed above [29].

As agglutinated bacteria are thought to resemble microcolonies formed *in vivo* [30] the bacterial cultures were observed using light microscopy (Fig. 6B). Whereas the bacteria were uniformly distributed in those cultures that also appeared homogenously at the macroscopic scale (Fig. 6A) the bacterial aggregates of strain AΔctxAB-[P_T7_]-tcp-T7RNAP grown in the presence of inducer were reminiscent of microcolonies (Fig. 6B) and very similar in appearance to previously published studies [32].

**Figure 7:**
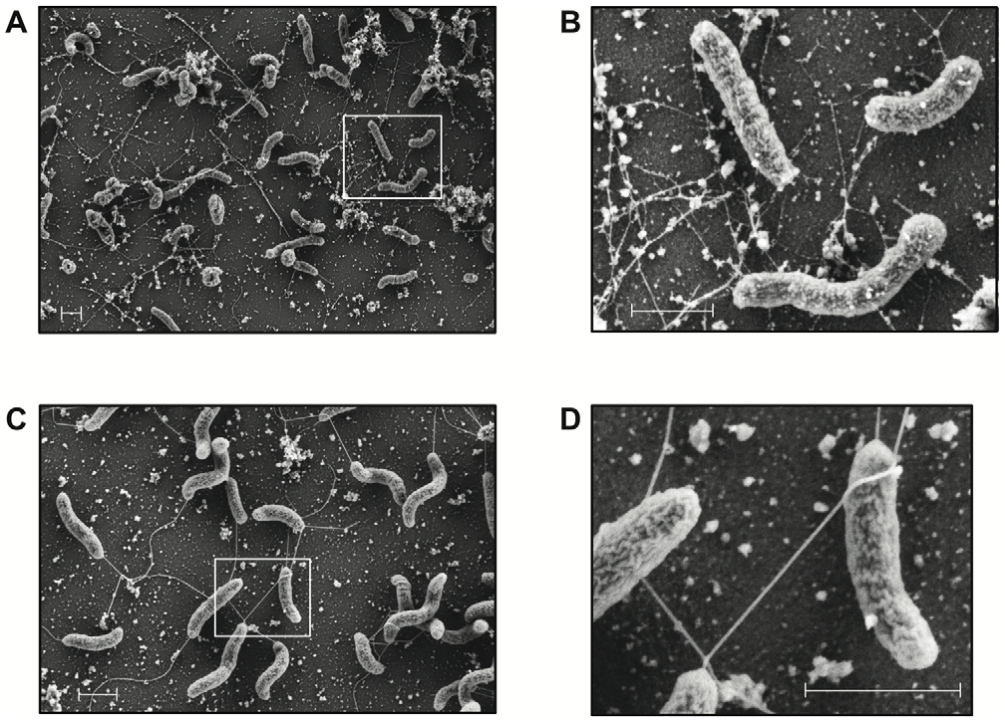
Visualization of TCP fibers by scanning electron microscopy. *V. cholerae* cells were grown in rich medium as described for Fig. 6. At that stage bacteria were transferred to silicon wafers and processed for SEM. A representative image of *V. cholerae* strain AΔctxAB-[P_T7_]-tcp-T7RNAP containing both the T7 RNA polymerase gene and P_T7_ promoter preceding the *tcp* cluster is shown in panel A (EHT = 2.00 kV, WD  = 3.7 mm, Mag  = 13.48 k X). The control strain lacking the P_T7_ promoter sequence upstream the *tcp* cluster is shown in panel C (EHT = 2.00 kV, WD  = 3.8 mm, Mag  = 24.91 k X). The white rectangles in panel A and C indicate the regions that are magnified in panels B and D, respectively. Scale bar  = 1 μm.

For visualization of the TCP pili using SEM (Fig. 7), the bacterial cells were grown as described above and as illustrated in Fig. 6. Subsequently, the bacteria were allowed to attach to a silicon wafer before being processed for SEM. It should be noted that the macroscopic clumps observed in Fig. 6A were partly flushed from the surface upon immersion of the silicon wafer into the fixative. This is consistent with the TCP pilus being involved in cell-to-cell interactions more so than for attachment to the silicon wafer. Nevertheless, enough bacteria were retained on the surface to allow visualization the TCP fibers (Fig. 7). Indeed, a plethora of TCP fibers were visible in the T7RNAP^+^ and P_T7_-carrying strain (Fig. 7A+B), whereas no such fibers were visible in a strain lacking the P_T7_ promoter upstream *tcpA* (Fig. 7C+D). Interestingly, Jude and Taylor have recently demonstrated that *V. cholerae* O1 El Tor derived TCP pili are thinner in width than classical TCP pili [32], which is in accordance with the thin TCP fibers we observed in our SEM images (Fig. 7A+B).

## Conclusions

In conclusion we successfully used the T7 RNA polymerase/promoter system in *V. cholerae*. To our knowledge this is the first time that this system was utilized in this organism though it was already suggested in earlier studies that “comparable T7 expression systems [to those in *E. coli*] can be developed in other types of cell” [12]. The advantage of this system is that the T7 RNA polymerase is highly selective for transcription from own promoters. A disadvantage that we encountered here and which was already reported earlier is that the lacUV5 promoter is not completely tight and thus leads to low expression of the T7 RNA polymerase even in the absence of inducer [12]. However, this problem has been addressed by others and several options to either tighten the promoter (e.g. using Lac repressor variants and/or production of increased levels of the repressor) or to reduce basal activity of undesired T7 RNA polymerase [43] have been suggested. As a next step we will exchange the lacUV5 promoter preceeding the T7 *gene 1* for the arabinose-inducible P_BAD_ promoter [44], which we have used with great success in earlier studies [7]. Judson and Mekalanos also used the P_BAD_ promoter in *V. cholerae* [45]. In their study P_BAD_ was outward-facing from a mariner-based transposon and as such used to transcriptionally fuse the promoter to different neighboring genes within the transposon library [45]. Here, we did not use random transposon insertion but instead we demonstrated target-specific gene expression as the T7-specific promoter was integrated by natural transformation and thus in a site-directed manner.

The data provided here demonstrate that the strategy depicted in Fig. 1 is functional. Thus, the combination of T7 RNA polymerase and the integration of the T7 RNA polymerase specific promoter sequence using natural transformation and FLP recombination (TransFLP) seems to be a promising tool to artificially induce gene clusters in *V. cholerae*.

## Supporting Information

Table S1 **Primers used in this study.**
(DOCX)

## References

[1] Sambrook J, Fritsch EF, Maniatis T (1982) Molecular Cloning: A Laboratory Manual. Cold Spring Harbor, NY.: Cold Spring Harbor Laboratory Press.

[2] Marvig RL, Blokesch M (2010) Natural transformation of *Vibrio cholerae* as a tool-optimizing the procedure. BMC Microbiol 10: 155. 20509862 10.1186/1471-2180-10-155 PMC2890613

[3] De Souza Silva O, Blokesch M (2010) Genetic manipulation of *Vibrio cholerae* by combining natural transformation with FLP recombination. Plasmid 64: 186–195. 20709100 10.1016/j.plasmid.2010.08.001

[4] Blokesch M (2012) TransFLP – a method to genetically modify *V. cholerae* based on natural transformation and FLP-recombination. J Vis Exp 68: e3761 doi:3710.3791/3761. 10.3791/3761 PMC3490321 23093249

[5] Meibom KL, Blokesch M, Dolganov NA, Wu C-Y (2005) Schoolnik GK (2005) Chitin induces natural competence in *Vibrio cholerae* . Science 310: 1824–1827. 16357262 10.1126/science.1120096

[6] Blokesch M (2007) Schoolnik GK (2007) Serogroup Conversion of *Vibrio cholerae* in Aquatic Reservoirs. PLoS Pathog 3: e81. 17559304 10.1371/journal.ppat.0030081 PMC1891326

[7] Lo Scrudato M, Blokesch M (2012) The Regulatory Network of Natural Competence and Transformation of *Vibrio cholerae* . PLoS Genet 8: e1002778. 22737089 10.1371/journal.pgen.1002778 PMC3380833

[8] Blokesch M (2012) A quorum sensing-mediated switch contributes to natural transformation of *Vibrio cholerae* Mob Genet Elements 2:5, Sep/Oct 2012; *published online first*. 10.4161/mge.22284 PMC3575429 23446800

[9] Seitz P, Blokesch M (2012) Cues and regulatory pathways involved in natural competence and transformation in pathogenic and environmental Gram-negative bacteria. FEMS Microbiol Rev Aug 28: doi: 10.1111/j.1574-6976.2012.00353.x. 10.1111/j.1574-6976.2012.00353.x 22928673

[10] Suckow G, Seitz P, Blokesch M (2011) Quorum Sensing Contributes to Natural Transformation of *Vibrio cholerae* in a Species-Specific Manner. J Bacteriol 193: 4914–4924. 21784943 10.1128/JB.05396-11 PMC3165701

[11] Tabor S, Richardson CC (1985) A bacteriophage T7 RNA polymerase/promoter system for controlled exclusive expression of specific genes. Proc Natl Acad Sci U S A 82: 1074–1078. 3156376 10.1073/pnas.82.4.1074 PMC397196

[12] Studier FW, Moffatt BA (1986) Use of bacteriophage T7 RNA polymerase to direct selective high-level expression of cloned genes. J Mol Biol 189: 113–130. 3537305 10.1016/0022-2836(86)90385-2

[13] Davanloo P, Rosenberg AH, Dunn JJ, Studier FW (1984) Cloning and expression of the gene for bacteriophage T7 RNA polymerase. Proc Natl Acad Sci USA 81: 2035–2039. 6371808 10.1073/pnas.81.7.2035 PMC345431

[14] Oakley JL, Coleman JE (1977) Structure of a promoter for T7 RNA polymerase. Proceedings of the National Academy of Sciences of the United States of America 74: 4266–4270. 270669 10.1073/pnas.74.10.4266 PMC431920

[15] Rosa MD (1979) Four T7 RNA polymerase promoters contain an identical 23 bp sequence. Cell 16: 815–825. 455451 10.1016/0092-8674(79)90097-7

[16] Panayotatos N, Wells RD (1979) Recognition and initiation site for four late promoters of phage T7 is a 22-base pair DNA sequence. Nature 280: 35–39. 15305578 10.1038/280035a0

[17] Ikeda RA, Ligman CM, Warshamana S (1992) T7 promoter contacts essential for promoter activity in vivo. Nucleic Acids Res 20: 2517–2524. 1598210 10.1093/nar/20.10.2517 PMC312387

[18] Dunn JJ, Studier FW (1983) Complete nucleotide sequence of bacteriophage T7 DNA and the locations of T7 genetic elements. Journal of molecular biology 166: 477–535. 6864790 10.1016/s0022-2836(83)80282-4

[19] Taylor RK, Miller VL, Furlong DB, Mekalanos JJ (1987) Use of *phoA* gene fusions to identify a pilus colonization factor coordinately regulated with cholera toxin. Proc Natl Acad Sci USA 84: 2833–2837. 2883655 10.1073/pnas.84.9.2833 PMC304754

[20] Karaolis DKR, Johnson JA, Bailey CC, Boedeker EC, Kaper JB, et al (1998) A *Vibrio cholerae* pathogenicity island associated with epidemic and pandemic strains. Proc Natl Acad Sci USA 95: 3134–3139. 9501228 10.1073/pnas.95.6.3134 PMC19707

[21] Brown RC, Taylor RK (1995) Organization of *tcp*, *acf*, and *toxT* genes within a ToxT-dependent operon. Mol Microbiol 16: 425–439. 7565104 10.1111/j.1365-2958.1995.tb02408.x

[22] Iwanaga M, Yamamoto K, Higa N, Ichinose Y, Nakasone N, et al (1986) Culture conditions for stimulating cholera toxin production by *Vibrio cholerae* O1 El Tor. Microbiol Immunol 30: 1075–1083. 3543624 10.1111/j.1348-0421.1986.tb03037.x

[23] Medrano AI, DiRita VJ, Castillo G, Sanchez J (1999) Transient transcriptional activation of the *Vibrio cholerae* El Tor virulence regulator toxT in response to culture conditions. Infect Immun 67: 2178–2183. 10225872 10.1128/iai.67.5.2178-2183.1999 PMC115955

[24] Herrington DA, Hall RH, Losonsky G, Mekalanos JJ, Taylor RK, et al (1988) Toxin, toxin-coregulated pili, and the *toxR* regulon are essential for *Vibrio cholerae* pathogenesis in humans. J Exp Med 168: 1487–1492. 2902187 10.1084/jem.168.4.1487 PMC2189073

[25] Tacket CO, Taylor RK, Losonsky G, Lim Y, Nataro JP, et al (1998) Investigation of the roles of toxin-coregulated pili and mannose-sensitive hemagglutinin pili in the pathogenesis of *Vibrio cholerae* O139 infection. Infect Immun 66: 692–695. 9453628 10.1128/iai.66.2.692-695.1998 PMC107958

[26] Thelin KH, Taylor RK (1996) Toxin-coregulated pilus, but not mannose-sensitive hemagglutinin, is required for colonization by *Vibrio cholerae* O1 El Tor biotype and O139 strains. Infect Immun 64: 2853–2856. 8698524 10.1128/iai.64.7.2853-2856.1996 PMC174155

[27] Krebs SJ, Taylor RK (2011) Protection and attachment of *Vibrio cholerae* mediated by the toxin-coregulated pilus in the infant mouse model. J Bacteriol 193: 5260–5270. 21804008 10.1128/JB.00378-11 PMC3187450

[28] Li J, Egelman EH, Craig L (2012) Structure of the *Vibrio cholerae* Type IVb Pilus and stability comparison with the Neisseria gonorrhoeae type IVa pilus. J Mol Biol 418: 47–64. 22361030 10.1016/j.jmb.2012.02.017 PMC3389824

[29] DiRita VJ, Neely M, Taylor RK, Bruss PM (1996) Differential expression of the ToxR regulon in classical and El Tor biotypes of *Vibrio cholerae* is due to biotype-specific control over *toxT* expression. Proc Natl Acad Sci USA 93: 7991–7995. 8755590 10.1073/pnas.93.15.7991 PMC38862

[30] Kirn TJ, Lafferty MJ, Sandoe CM, Taylor RK (2000) Delineation of pilin domains required for bacterial association into microcolonies and intestinal colonization by *Vibrio cholerae* . Mol Microbiol 35: 896–910. 10692166 10.1046/j.1365-2958.2000.01764.x

[31] Lim MS, Ng D, Zong Z, Arvai AS, Taylor RK, et al (2010) *Vibrio cholerae* El Tor TcpA crystal structure and mechanism for pilus-mediated microcolony formation. Mol Microbiol 77: 755–770. 20545841 10.1111/j.1365-2958.2010.07244.x PMC2939829

[32] Jude BA, Taylor RK (2011) The physical basis of type 4 pilus-mediated microcolony formation by *Vibrio cholerae* O1. J Struct Biol 175: 1–9. 21527347 10.1016/j.jsb.2011.04.008 PMC3102138

[33] Matson JS, Withey JH, DiRita VJ (2007) Regulatory networks controlling *Vibrio cholerae* virulence gene expression. Infect Immun 75: 5542–5549. 17875629 10.1128/IAI.01094-07 PMC2168339

[34] Yanisch-Perron C, Vieira J, Messing J (1985) Improved M13 phage cloning vectors and host strains: nucleotide sequences of the M13mp18 and pUC19 vectors. Gene 33: 103–119. 2985470 10.1016/0378-1119(85)90120-9

[35] Simon R, Priefer U, Pühler A (1983) A Broad Host Range Mobilization System for *In Vivo* Genetic Engineering: Transposon Mutagenesis in Gram Negative Bacteria. Nat Biotechnol 1: 784–791.

[36] Blokesch M (2012) Chitin colonization, chitin degradation and chitin-induced natural competence of *Vibrio cholerae* are subject to catabolite repression. Environ Microbiol 14: 1898–1912. 22222000 10.1111/j.1462-2920.2011.02689.x

[37] Bao Y, Lies DP, Fu H, Roberts GP (1991) An improved Tn*7*-based system for the single-copy insertion of cloned genes into chromosomes of gram-negative bacteria. Gene 109: 167–168. 1661697 10.1016/0378-1119(91)90604-a

[38] Lambertsen L, Sternberg C, Molin S (2004) Mini-Tn*7* transposons for site-specific tagging of bacteria with fluorescent proteins. Environ Microbiol 6: 726–732. 15186351 10.1111/j.1462-2920.2004.00605.x

[39] Beyhan S, Tischler AD, Camilli A, Yildiz FH (2006) Transcriptome and phenotypic responses of Vibrio cholerae to increased cyclic di-GMP level. J Bacteriol 188: 3600–3613. 16672614 10.1128/JB.188.10.3600-3613.2006 PMC1482859

[40] Chamberlin M, McGrath J, Waskell L (1970) New RNA polymerase from *Escherichia coli* infected with bacteriophage T7. Nature 228: 227–231. 4920917 10.1038/228227a0

[41] Nielsen AT, Dolganov NA, Rasmussen T, Otto G, Miller MC, et al (2010) A Bistable Switch and Anatomical Site Control *Vibrio cholerae* Virulence Gene Expression in the Intestine. PLoS Pathog 6: e1001102. 20862321 10.1371/journal.ppat.1001102 PMC2940755

[42] Yildiz FH (1998) Schoolnik GK (1998) Role of *rpoS* in stress survival and virulence of *Vibrio cholerae* . J Bacteriol 180: 773–784. 9473029 10.1128/jb.180.4.773-784.1998 PMC106954

[43] Studier FW (1991) Use of bacteriophage T7 lysozyme to improve an inducible T7 expression system. J Mol Biol 219: 37–44. 2023259 10.1016/0022-2836(91)90855-z

[44] Schleif R (2010) AraC protein, regulation of the l-arabinose operon in *Escherichia coli*, and the light switch mechanism of AraC action. FEMS Microbiol Rev 34: 779–796. 20491933 10.1111/j.1574-6976.2010.00226.x

[45] Judson N, Mekalanos JJ (2000) TnAraOut, A transposon-based approach to identify and characterize essential bacterial genes. Nat Biotechnol 18: 740–745. 10888841 10.1038/77305

[46] Faruque SM, Zhu J, Asadulghani, Kamruzzaman M, Mekalanos JJ (2003) Examination of diverse toxin-coregulated pilus-positive *Vibrio cholerae* strains fails to demonstrate evidence for *Vibrio* pathogenicity island phage. Infect Immun 71: 2993–2999. 12761075 10.1128/IAI.71.6.2993-2999.2003 PMC155729

[47] Heidelberg JF, Eisen JA, Nelson WC, Clayton RA, Gwinn ML, et al (2000) DNA sequence of both chromosomes of the cholera pathogen *Vibrio cholerae* . Nature 406: 477–483. 10952301 10.1038/35020000 PMC8288016

