# Supplementary material for: Overexpression of the tcp Gene Cluster Using the T7 RNA Polymerase/Promoter System and Natural Transformation-Mediated Genetic Engineering of Vibrio cholerae
Source: PLoS One. 2013 Jan 7;8(1):e53952. doi: 10.1371/journal.pone.0053952 (PMC3538720; doi:10.1371/journal.pone.0053952)
Supplement: Table S1 — Primers used in this study. (DOCX) [file pone.0053952.s001.docx]

**TABLE S1. Primers used in this study**

| **Primer name** | **Sequence***  (given in 5’ to 3’ direction) | **Comments** |
| --- | --- | --- |
| P[T7]-GFP-up-P | GAAATTAATACGACTCACTATAGGGAGACACTCTAGAATTAAAGAGGAG | Amplification of *gfp* concomitantly adding T7 RNA-polymerase dependent promoter (underlined); template: gDNA of strain A1552-GFP [[36](#_ENREF_36)] |
| P[T7]-GFP-down-BamHI | CGC*GGATCC*AGCTTATTTGTATAGTTCATCCATGCC |  |
| LacI-before | CCC*AGTACT*CCAGATCCCGGACACCATCGAATGGCG | Amplification of *lacI,* P_lacUV5_ and the gene encoding the T7 RNA polymerase to clone into plasmid pGP704::Tn7 |
| T7 RNA pol after | CCC*AGTACT*TATGCGCGCACGAAAAGCATCAGGTC |  |
| CtxAB-FRT-Kan#1 | CAGCCTTACAATAGAAATAAATCAAGC | To create transforming PCR fragment ΔctxAB-FRT-Kan-FRT (according to TransFLP method described by [[3](#_ENREF_3),[4](#_ENREF_4)]) |
| CtxAB-FRT-Kan#2 | AGCTCCAGCCTACACTTCTGATTCCAGCCGTCCGCCGC |  |
| CtxAB-FRT-Kan#3 | TGGAATCAGAAGTGTAGGCTGGAGCTGCTTCGAAGTTCC |  |
| CtxAB-FRT-Kan#4 | GAGCATTATCATATGAATATCCTCCTTAGTTCCTATTCC |  |
| CtxAB-FRT-Kan#5 | AAGGAGGATATTCATATGATAATGCTCCCTTTGTTTAAC |  |
| CtxAB-FRT-Kan#6 | CTGGACAGATGGACTCGCACGCGCTGAC |  |
| CtxAB-check-up | ACGGAGAAGCGTTTGTATCGAGTTG | To check correct DNA exchange and FLP-mediated excision at the *ctxAB* locus of naturally transformed *V. cholerae* cells |
| CtxAB-check-down | GCCAAAGTGCACAACATGATAAGAG |  |
| T7tcp_1 | ACAATGATTATTGATGGTACAGTCACAC | To create transforming PCR fragment (as illustrated in Fig. 4) for TransFLP-mediated genome engineering |
| T7tcp_2 | CAGCTCCAGCCTACGCTAGAAAAAGAAAGCAATACGCAC |  |
| T7tcp_3 | TTCTTTTTCTAGCGTAGGCTGGAGCTGCTTCGAAGTTCC |  |
| T7tcp_4 | TCTCCCTATAGTGAGTCGTATTAATTTCCATATGAATATCCTCCTTAGTTCCTATTC |  |
| T7tcp_5 | GAAATTAATACGACTCACTATAGGGAGATTAAAAAAGGACCAAGCAATGCATTTCC |  |
| T7tcp_6 | TTCCTGTACAAAGCTTCTCAACATGCG |  |
| T7tcp_chk-up | GCTACAGGTCTATTCGTTGTAGCAC | To check correct DNA exchange and FLP-mediated excision around the *tcpA* gene of naturally transformed bacteria |
| T7tcp_chk-down | ATCACTTTTAGCTTAATAATTTGCC |  |
| gyrA-157-fwd | AATGTGCTGGGCAACGACTG | qRT-PCR for *gyrA* transcription |
| gyrA_332_bwd | GAGCCAAAGTTACCTTGGCC |  |
| qRT_tcpI_fwd | AGTTGCGTTGTTGCTGTGGC | qRT-PCR for *tcpI* transcription |
| qRT_tcpI_bwd | ACGAGCTCGACACTATTGCC |  |
| qRT_tcpH_fwd | TTGGCTTACCCAGACCGATC | qRT-PCR for *tcpH* transcription |
| qRT_tcpH_bwd | AGCAGCTTGTAAGGGAAGGC |  |
| qRT tcpA for | GAATATGACTAAGGCTGCGC | qRT-PCR for *tcpA* transcription |
| qRT tcpA rev | GCTGAAACCTTACCAAGGCT |  |
| qRT_tcpB_fwd | TCAGTCTTGCCCAAACCGGA | qRT-PCR for *tcpB* transcription |
| qRT_tcpB_bwd | ATAGGCCTTTCGCACTGACC |  |
| qRT_tcpC_fwd | ATACTCAGGTCCTCTGGCTG | qRT-PCR for *tcpC* transcription |
| qRT_tcpC_bwd | CTTGATGTGCCACTGGTACC |  |
| qRT_tcpD_fwd | AGGTCTTGATTACCAAGCGC | qRT-PCR for *tcpD* transcription |
| qRT_tcpD_bwd | AAGGTGTCAAAGCTTCCTCG |  |
| qRT_tcpT_fwd | ATTCGATGAAGAGCGTGAGG | qRT-PCR for *tcpT* transcription |
| qRT_tcpT_bwd | CTGCTCGGTAACAAGATTCG |  |
| qRT_tcpF_fwd | GAAGCTACGGATTCAAGGGG | qRT-PCR for *tcpF* transcription |
| qRT_tcpF_bwd | AGCACCGTCAGATTCTGTCG |  |
| qRT_toxT_fwd | TACGCGTAATTGGCGTTGGG | qRT-PCR for *toxT* transcription |
| qRT_toxT_bwd | ACGCTAGCAAACCCAGACTG |  |
| qRT_acfB_fwd | TTCGATCCAAGGACTCGATC | qRT-PCR for *acfB* transcription |
| qRT_acfB_bwd | TCCTGAACGTCATCTAGTGG |  |
| qRT_acfD_fwd | GCCAGATATTGGTGTTCCGG | qRT-PCR for *acfD* transcription |
| qRT_acfD_bwd | GATTGAACGCCCGTTGATCG |  |
| qRT_recA_fwd | CTCTGTCTCTGGATATCGCG | qRT-PCR for *recA* transcription |
| qRT_recA_bwd | GCATAAACCGGATCCAGTGC |  |

* 5’ added restriction enzyme recognition sites are indicated in italics
